# Safety Checklist Implementation Did Not Reduce Central Venous Catheter Duration in Pediatric Cardiac ICU Patients

**DOI:** 10.1097/pq9.0000000000000253

**Published:** 2020-01-22

**Authors:** Raj Sahulee, Michelle M. Ramirez, Yasir M. Al-Qaqaa, Sujata B. Chakravarti, Jaclyn McKinstry

**Affiliations:** From the * Department of Pediatrics, Division of Cardiology, NYU School of Medicine, New York, N.Y.; † Department of Pediatrics, Division of Critical Care Medicine, NYU School of Medicine, New York, N.Y.

## Abstract

The Center for Disease Control recommends prompt removal of nonessential central venous catheters (CVCs) to reduce the risk for central line–associated bloodstream infections. Safety checklists have been trialed to reduce nonessential CVC days, but pediatric studies are lacking. Our specific aim was to detect >10% reduction in mean CVC duration after implementation of a safety checklist addressing CVCs in our unit.

**Methods:**

All patients admitted to the Congenital Cardiovascular Care Unit at New York University Langone Medical Center who had a CVC placed between January 1, 2012, and December 31, 2017, were included. We implemented a checklist addressing CVC use in our unit on June 7, 2013, and modified it on March 10, 2016. We analyzed quarterly mean CVC duration and postsurgical CVC duration over the study period using statistical process control charts.

**Results:**

We placed 778 CVCs for 7,947 CVC days during the study period. We noted special cause variation from Q4 2013 to Q2 2014 and a centerline shift in mean CVC duration from 8.91 to 11.10 days in Q1 2015. In a subgroup analysis of the 657 lines placed in surgical patients, there was a centerline shift in mean CVC duration from 6.48 to 8.86 days in Q4 2013.

**Conclusions:**

Our study demonstrated an unexpected increase in mean CVC duration after the implementation of a safety checklist designed to decrease nonessential CVC days. Additional studies are needed to identify the ideal method to detect and remove nonessential CVCs and reduce the risk of preventable harm.

## INTRODUCTION

Central venous catheters (CVCs) are often essential in the care of critically ill children. However, their use carries risks for catheter-related adverse events (CR-AEs) such as central line–associated bloodstream infection (CLABSI) and venous thromboembolism.^1,2^ Considerable effort has been dedicated to eliminating these CR-AEs. To reduce the incidence of CLABSI, the Center for Disease Control (CDC) recommends the removal of nonessential CVCs.^3^ However, no consensus definition of an “essential” CVC currently exists, nor are there published guidelines for their removal. Not unexpectedly, studies have shown that this recommendation by the CDC is poorly followed^4,5^ and may account for much of the CLABSI variability between centers.^5^

Although prompt removal of nonessential CVCs is recommended, the ideal method to reliably detect and remove unnecessary CVCs is unknown. Importantly, some reports indicate that 15%−28% of all CVC days are “idle” or nonessential.^6–8^ Furthermore, up to 63% of all patients who have a CVC placed will experience at least 1 idle CVC day.^8,9^ Removing nonessential CVCs can reduce patients’ risk for CR-AE. These studies highlight the gap between current and recommended practice in efforts to eliminate preventable patient harm.

Various methods have been trialed to identify and remove nonessential CVCs with limited success.^4,10–12^ Safety checklists are frequently used to implement best practices that help prevent CR-AE and other hospital-acquired conditions. Theoretically, when providers consistently utilize a properly designed safety checklist, they would identify and promptly remove nonessential CVCs. As a result, total CVC days and cumulative patient risk for CR-AE should decrease over time. Although multifaceted strategies for CLABSI reduction have been well described,^10,13^ a knowledge gap remains on how to reduce nonessential catheter-days effectively,^13–15^ especially for children.^16^ Therefore, we aimed for a >10% reduction in mean CVC duration within 1 year of implementing a daily safety checklist that addressed the ongoing need for a CVC in our Congenital Cardiovascular Care Unit (CCVCU).

## METHODS

We performed a retrospective review of all patients admitted to the CCVCU at Hassenfeld Children’s Hospital at New York University (NYU) Langone Medical Center, who had a CVC placed between January 1, 2012, and December 31, 2017. For this study, we defined a CVC as any percutaneously placed or tunneled CVC, peripherally inserted central catheter, or umbilical venous catheter. The CCVCU at NYU New York University Langone Medical Center cares for patients from birth to 24 years of age with various forms of congenital and acquired heart disease requiring medical or surgical intensive care. Of note, during the study, we did not offer heart transplantation services, nor did the CCVCU operate as an admit-to-discharge unit.

**Fig. 1.**
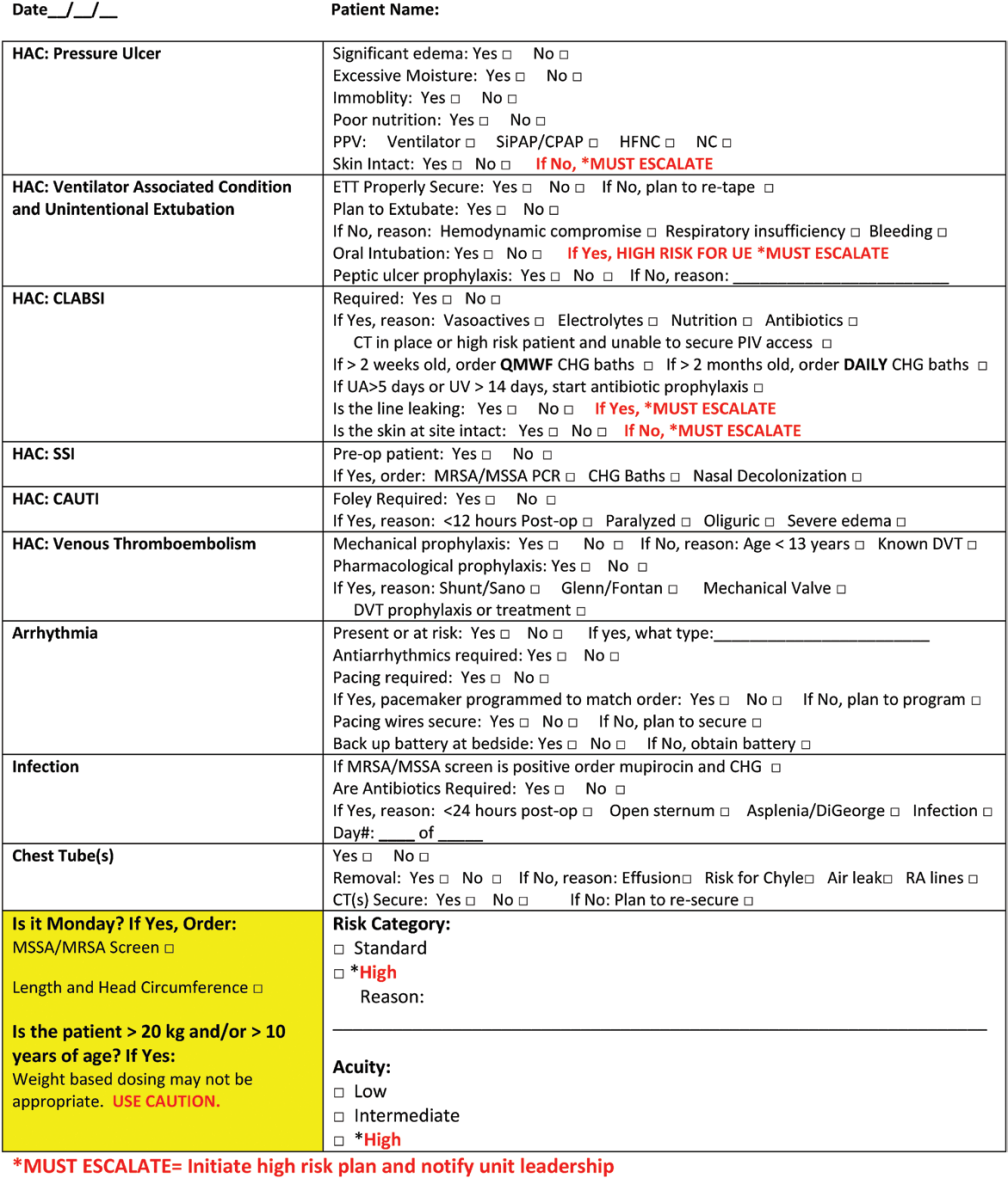
CCVCU interdisciplinary safety checklist (to be completed during daily work rounds). *MUST ESCALATE = initiate high-risk plan and notify unit leadership. CT indicates computed tomography. HAC, hospital-acquired condition; PPV, positive pressure ventilation; SiPAP, synchronized intermittent positive airway pressure; CPAP, continuous positive airway pressure; HFNC, high flow nasal cannula; NC, nasal cannula; ETT, endotracheal tube; UE, unplanned extubation; CT, chest tube; PIV, peripheral intravenous catheter; QMWF, every Monday, Wednesday and Friday; CHG, chlorhexadine gluconate; UA, umbilical artery catheter; UV, umbilical venous catheter; SSI, surgical site infection; MRSA, methicillin-resistant *Staphylococcus aureus*; MSSA, Methicillin-sensitive *Staphylococcus aureus*; PCR, polymerase chain reaction; CAUTI, catheter-associated urinary tract infection; DVT, deep vein thrombosis; RA, right atrial.

Medical, surgical, and nursing leadership designed a 1-page safety checklist to reduce the incidence of CLABSI and other hospital-acquired conditions in the CCVCU (Fig. 1). We first implemented the daily safety checklist on June 7, 2013. We modified the checklist on March 10, 2016, to include the CVC indication (or necessity) at that time. As stated previously, there is not a universally accepted definition of an “essential” CVC from the CDC or other healthcare governing body. Therefore, we defined an essential CVC as one used for administering vasoactive medications, prolonged antibiotics, total parenteral nutrition, or electrolyte replacements. We also deemed CVCs placed in high-risk patients without peripheral intravenous access as essential. We utilized the safety checklist while at the bedside of each patient at the end of daily rounds, with the patient and parents when available, and before progressing to the next patient. Any of the providers in the care team (physicians, charge nurses, nurse practitioners, or cardiology fellows) could administer the checklist. If a safety concern was found, the entire team huddled and discussed any required interventions. Specifically for CVCs, if the team identified a nonessential CVC, a discussion about CVC removal was to be held. Finally, after the completion of daily rounds, nursing leadership collected the checklists and later analyzed them for compliance monitoring.

We included all CCVCU patients who had a CVC in our sample and performed a separate analysis of the surgical cohort. First, we compared patient demographics and catheter details between the cohorts from 2012–2014 and 2015–2017 using the chi-square test, *t* test, and Mann-Whitney *U* test. Next, we calculated the mean CVC duration on a quarter-yearly basis throughout the study period. Then we used statistical process control to generate X-bar/S-charts to evaluate for changes in quarterly mean CVC duration and postsurgical CVC duration. Due to the rightward skew of CVC days in the patient population, we used a (b + ax)^1/3^ transformation to create the upper and lower control limits per quarter. We evaluated for special cause variation and trends or shifts of the centerline for mean CVC duration using the Nelson rules.^17^ Finally, we recorded compliance with the completion of the checklist on each patient by quarter. Statistical analysis was performed using IBM SPSS version 23 (IBM Corporation, Armonk, N.Y.) and QI Macros (KnowWare International, Inc., Denver, Colo.).

## RESULTS

**Fig. 2.**
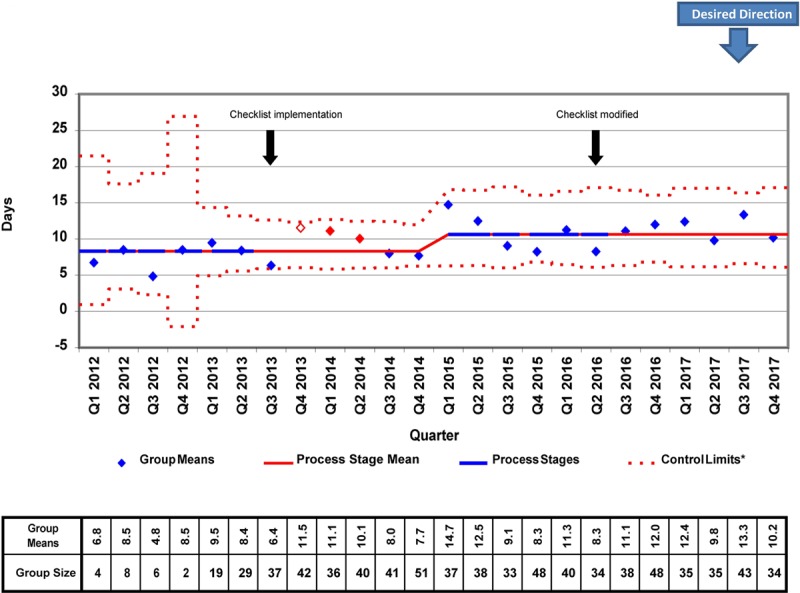
Total CVC days X-bar chart. *A (b + ax)^1/3^ transform to correct for right skew was used to determine control limits. Limits were then reverse transformed to reflect original data metrics.

**Fig. 3.**
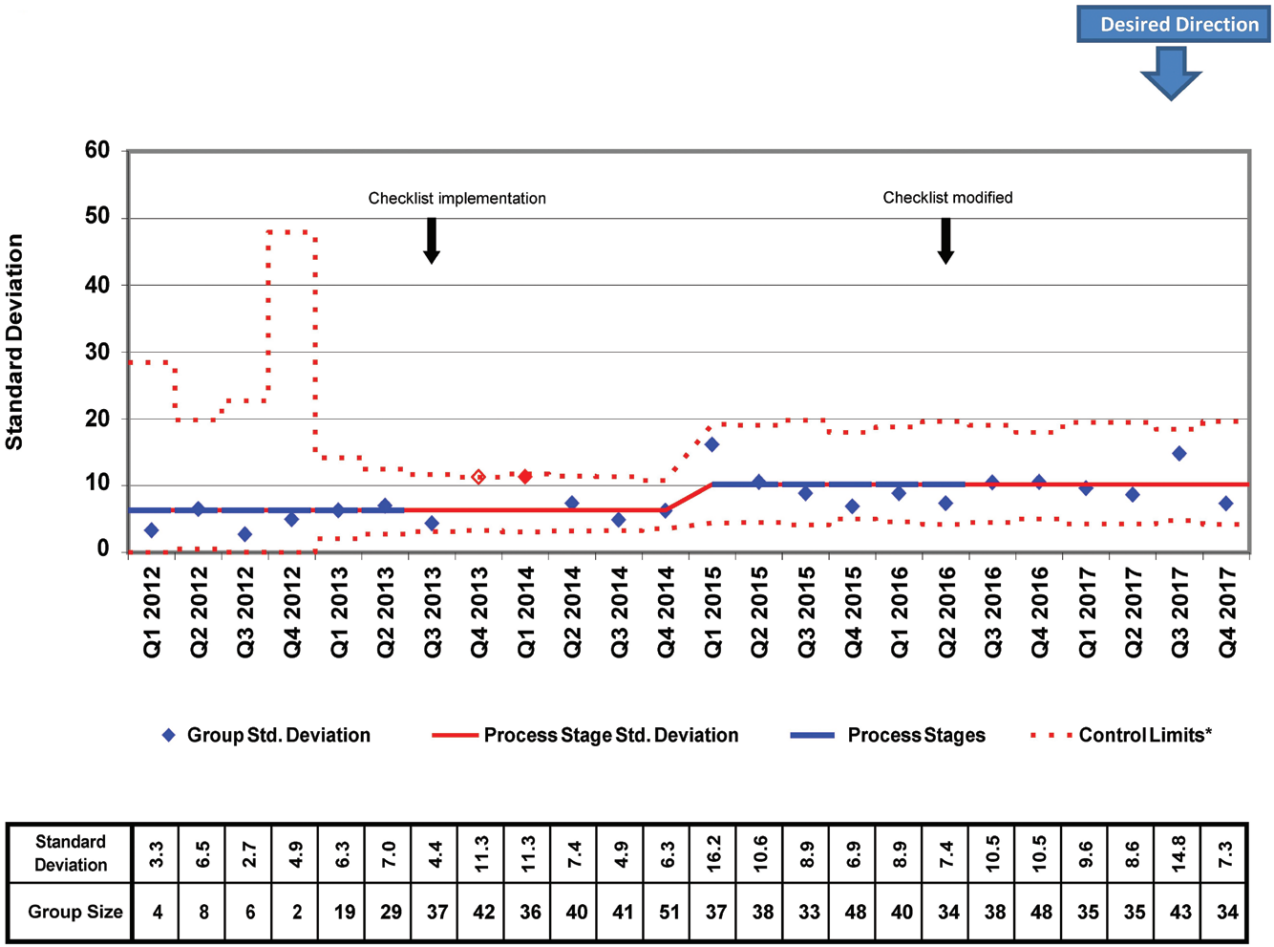
Total CVC days S-chart. *A (b + ax)^1/3^ transform to correct for right skew was used to determine control limits. Limits were then reverse transformed to reflect original data metrics.

**Fig. 4.**
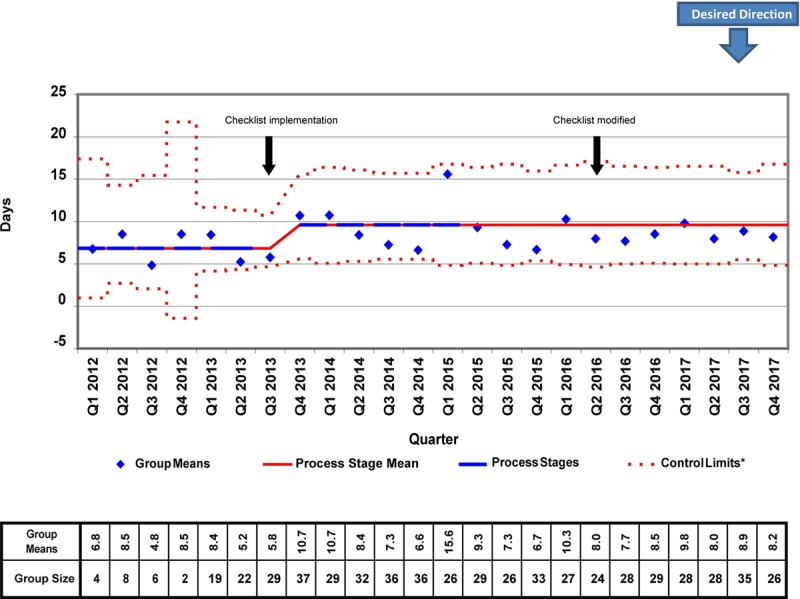
Postsurgical CVC days X-bar chart. *A (b + ax)^1/3^ transform to correct for right skew was used to determine control limits. Limits were then reverse transformed to reflect original data metrics.

**Fig. 5.**
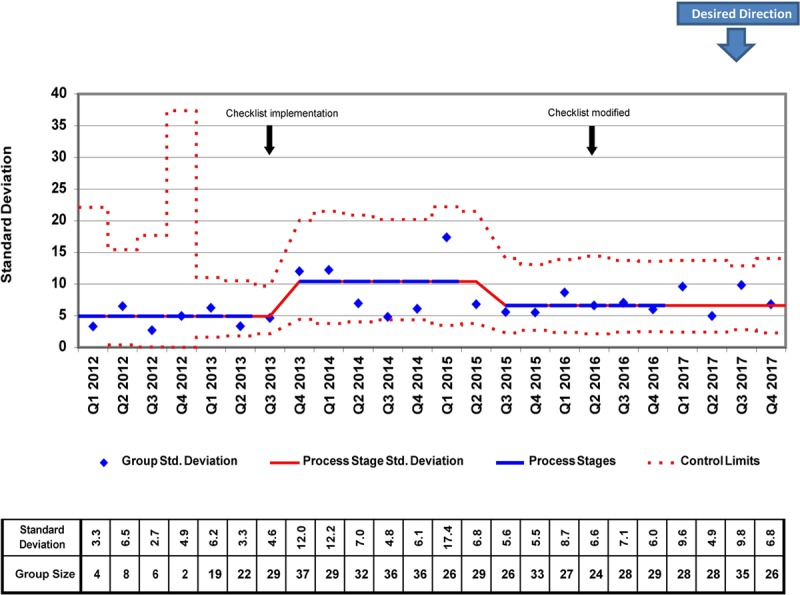
Postsurgical CVC days S-chart. *A (b + ax)^1/3^ transform to correct for right skew was used to determine control limits. Limits were then reverse transformed to reflect original data metrics.

We placed 778 CVCs for a total of 7947 CVC days during the study period. There were several differences in patient demographics and catheter details between the 2012−2014 and 2015−2017 cohorts, but only the proportion of surgical patients achieved statistical significance (87.38% versus 82.32%; *P* = 0.041; Table 1). In the analysis of the X-bar/S-charts for total CVC days, we noted special cause variation (Nelson rule #5) in Q4 2013, Q1 2014, and Q2 2014. Also, there was a centerline shift upwards (Nelson rule #2) in mean CVC duration from 8.91 to 11.10 days in Q1 2015 (Figs. 2, 3). In subgroup analysis, we placed 657 lines in surgical patients, and there was a centerline shift (Nelson rule #2) in mean CVC duration from 6.48 to 8.86 days in Q4 2013 without special cause variation noted (Figs. 4, 5). Overall compliance with completion of the safety checklist was high, ranging from 96.3% to 99.8% per quarter.

**Table 1.**
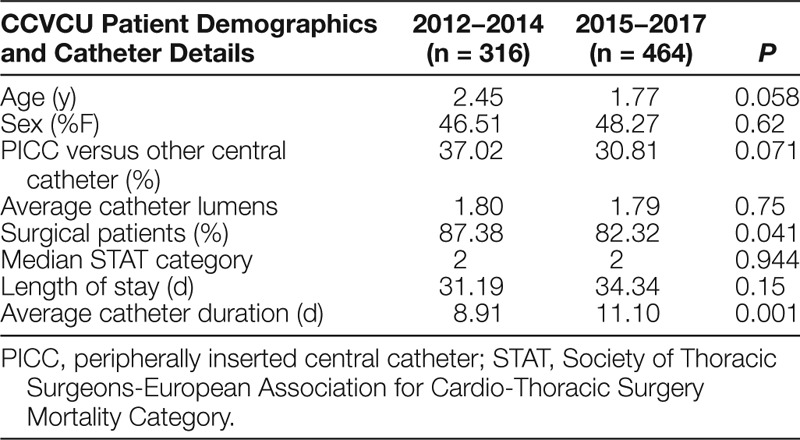
Patient Demographics and Catheter Details

## DISCUSSION

Our study demonstrated that the implementation of a daily safety checklist was not associated with a >10% decrease but rather an increase in the mean CVC duration and postsurgical CVC duration in our CCVCU. Our inability to demonstrate a significant reduction in CVC duration after implementation of a safety checklist is consistent with the data described by Pronovost et al^13^ in the Keystone ICU study. Their landmark study of 375,757 catheter-days in 103 adult ICUs failed to demonstrate a significant reduction in catheter-days after the implementation of a daily goal sheet.^13^ Although the efficacy of safety checklists to reduce CVC days is highly variable among adult studies,^4,10,14,15,18^ our study and others have been unable to demonstrate a reduction in catheter duration for pediatric patients specifically.^16^

Surprisingly, we observed a centerline shift toward longer mean CVC duration and postsurgical CVC duration following checklist implementation. The reasons for the shifts are unclear. When examining patient demographics, there was a significantly lower proportion of surgical patients from 2015–2017 when compared with 2012−2014. Therefore, it is possible that the increased CVC duration observed was related to the increased proportion of medical patients. Another possible explanation for this finding is that the absolute increase in the number of CVCs placed during the years 2015−2017. This difference increased the probability of having >1 patient with prolonged CVC days during the same quarter. This explanation is supported by the increase in the centerline of the average standard deviation in the total CVC days S-chart (Fig. 3). Furthermore, although not reaching statistical significance, there was a trend toward younger patients (1.77 versus 2.45 years; *P* = 0.058) in 2015−2017 versus 2012−2014. The illness severity of these younger patients may have increased the need for prolonged CVC use. Unfortunately, given the limitations of our CVC data, we had limited ability to assess for changes in illness severity during the study period objectively. However, with regards to our surgical subgroup, the median Society of Thoracic Surgeons-European Association for Cardio-Thoracic Surgery (STAT) mortality category, a marker of surgical risk and a possible surrogate for patient acuity, was not significantly different between the 2 cohorts. Despite these hypotheses, the observed increase in mean CVC duration remains challenging to explain. Regardless, our study and others demonstrate that we have yet to identify the ideal method to detect and remove nonessential CVCs to reduce total days at risk for CR-AE. However, it is important to note that several studies have successfully demonstrated that the use of multifaceted safety checklists is associated with improvements in outcome measures like CLABSI rates,^4,10,13^ even when process measures like mean catheter duration are not significantly improved.

There remains an epidemic of idle CVCs in healthcare. Recent studies have demonstrated that up to 28% of all CVC days are idle, 50%−63% of patients experience at least 1 idle day, and 38% experience 2 consecutive idle days.^6–9^ Although our study could not assess for changes in nonessential CVC days specifically, these studies suggest that mean CVC duration, and days at risk for CR-AE, could be significantly reduced if nonessential lines are removed in a timely fashion. As McLaws and Berry^19^ demonstrate, promptly removing nonessential CVCs is critical as CLABSI rates increase significantly with longer CVC use. Furthermore, Rotz and Sopirala^20^ found that 22% of CLABSIs occurred in patients with CVCs that were no longer indicated. In their multi-institutional study, Weeks et al^4^ demonstrated a modest 4% reduction in CVC duration after checklist implementation, far short of eliminating the estimated 15%−28% idle CVC days patients experience. The authors proposed that CVCs are often left in place as a matter of convenience for rapid medication or fluid administration, blood draws, and to diminish needle sticks. They also found that the removal of nonessential lines was one of the least used risk-reduction practices before checklist implementation. Similarly, Hsu et al^5^ demonstrated that removing nonessential CVCs had the lowest compliance among CLABSI prevention recommendations. Moreover, this behavior largely accounted for the variability in CLABSI rates among the centers involved in their study.^5^ A gap between current practice and recommended practice remains, so how do teams reliably identify and remove nonessential CVCs to minimize the risk of harm to our patients?

Removing nonessential CVCs requires teamwork among physicians, nurses, and other healthcare providers. Safety checklists and daily goal sheets are some of the most common tools used to reduce the risk of hospital-acquired conditions. Checklists are tools to ensure compliance with evidence-based practices, enhance communication, promote consistency of care, and can improve outcomes.^21^ However, the completion of a checklist does not necessarily translate to the prompt removal of nonessential CVCs. Ko et al^22^ demonstrated in their meta-analysis that the use of checklists is not consistently associated with improvement in care processes. They also found that there is rarely validation of the content in safety checklists, and guidelines for the removal of CVCs are lacking. Therefore, despite consistent utilization of the safety checklist in our study, the use of a nonvalidated definition of CVC necessity may have potentially contributed to the failure to reduce mean CVC duration for our patients.

As an alternative strategy to checklists, mandatory daily documentation of CVC necessity has been proposed as a method to reduce CVC days. However, compliance with daily documentation does not necessarily translate to the removal of nonessential catheters or reduce CVC duration.^11^ Dedicated interdisciplinary teams have also been trialed to aid in identifying and removing nonessential CVCs but have similarly demonstrated limited efficacy in reducing catheter-days.^12^ As these tools have yet to demonstrate a consistent reduction in CVC duration, we must consider if it is efficient to continue utilizing them. There are several balancing costs involved in implementing processes that are unable to improve patient safety. Daily safety checklists require provider time, training, and even potential costs in hiring additional staff to implement these safety tools. Therefore, to effectively reduce nonessential CVC days and patient risk, we must either develop an entirely novel approach or implement a combination of known tools.

There are several key limitations to this study. First, this study was a retrospective review of CVC duration in one unit at one institution. The study was limited to this design because the safety checklist was designed specifically for the needs of the patients in the CCVCU and, thus, was not implemented in other units for comparison. Second, we cannot prove that the increase in mean CVC duration was not due to a change in acuity of the patient population over time. During the study period, our patient volume was dramatically reduced during the 9-month near-total shutdown of the CCVCU following Hurricane Sandy in 2012. Thus, our patient and CVC volume was significantly less in the 2012−2014 cohort compared with 2015−2017. We also cared for a significantly higher proportion of nonsurgical patients in 2015−2017, further suggesting a possible change in our study population. Unfortunately, given the limits of our CVC data, we were unable to assess for changes in illness severity during the study objectively, a substantial limitation of our study. We also did not assess for unintended consequences of CVC removal, such as the need for catheter replacement. Finally, and most importantly, we were unable to assess the frequency of nonessential or “idle” CVC days. Given our data limitations, we could not temporally relate the use of the checklist with the timing of CVC removal and, therefore, chose to assess for changes in mean CVC duration.

In conclusion, to minimize the risks for CR-AE, we must identify and promptly remove CVCs that are no longer essential. The implementation of a daily safety checklist was not associated with a reduction in mean CVC duration in children with critical cardiac illness admitted to our CCVCU. Although safety checklists are one frequently utilized tool, further multifaceted efforts are needed to reduce days at risk for CR-AE as we strive to eliminate preventable harm for all children.

## ACKNOWLEDGMENTS

The authors thank Sarah Pender for her contribution to data collection.

## Disclosure:

The authors have no financial interest to declare in relation to the content of this article.
